# Evaluation of principal component analysis-based data-driven respiratory gating for positron emission tomography

**DOI:** 10.1259/bjr.20170793

**Published:** 2018-03-08

**Authors:** Matthew D Walker, Kevin M Bradley, Daniel R McGowan

**Affiliations:** 1 Radiation Physics and Protection, Churchill Hospital, Oxford University Hospitals NHS Foundation Trust, Oxford, UK; 2 Department of Radiology, Churchill Hospital, Oxford University Hospitals NHS Foundation Trust, Oxford, UK; 3 Department of Oncology, University of Oxford, Oxford, UK

## Abstract

**Objective:**

Respiratory motion can degrade PET image quality and lead to inaccurate quantification of lesion uptake. Such motion can be mitigated via respiratory gating. Our objective was to evaluate a data-driven gating (DDG) technique that is being developed commercially for clinical PET/CT.

**Methods:**

A data-driven respiratory gating algorithm based on principal component analysis (PCA) was applied to phantom and FDG patient data. An anthropomorphic phantom and a NEMA IEC Body phantom were filled with ^18^F, placed on a respiratory motion platform, and imaged using a PET/CT scanner. Motion waveforms were measured using an infrared camera [the Real-time Position Management™ system (RPM)] and also extracted from the PET data using the DDG algorithm. The waveforms were compared via calculation of Pearson’s correlation coefficients. PET data were reconstructed using quiescent period gating (QPG) and compared via measurement of recovery percentage and background variability.

**Results:**

Data-driven gating had similar performance to the external gating system, with correlation coefficients in excess of 0.97. Phantom and patient images were visually clearer with improved contrast when QPG was applied as compared to no motion compensation. Recovery coefficients in the phantoms were not significantly different between DDG- and RPM-based QPG, but were significantly higher than those found for no motion compensation (*p* < 0.05).

**Conclusion:**

A PCA-based DDG algorithm was evaluated and found to provide a reliable respiratory gating signal in anthropomorphic phantom studies and in example patients.

**Advances in knowledge:**

The prototype commercial DDG algorithm may enable reliable respiratory gating in routine clinical PET-CT.

## Introduction

Respiratory motion can significantly degrade PET image quality. The radioactivity distribution is blurred by the motion, leading to an image that may be suboptimal for tasks including diagnosis and quantitative assessment. Respiratory gating can be used to lessen the effect of motion, but requires acquisition of a gating signal.^1, 2^ Such signals can be obtained from external devices such as a video camera or pressure belt; these may be inconvenient or unavailable, but have been used with some success.^3, 4^ A gating signal can also be extracted directly from the acquired projection data using a variety of methods, commonly referred to as data-driven gating (DDG).^5–21^ The aim of the current investigation is to evaluate a DDG algorithm based on principal component analysis (PCA) which is currently being developed by GE for routine clinical use.^9, 10^ The evaluation is based on data from phantom experiments complemented by two patient examples.

As PET data are acquired for several minutes at each bed position, the images represent a time-averaged radioactivity distribution which is blurred by the many respiratory cycles if no compensation for respiratory motion is made. The amplitude of respiratory motion varies considerably for different organs and across different patients. The average motion in the cranio-caudal direction, which is the main direction of motion, is about 24 mm for the liver and pancreas, and about 17 mm for the kidneys.^22–24^ In addition to causing a blur in PET images, respiratory motion leads to a mis-match between PET and CT images in PET-CT scanning. This mis-match is the cause of common attenuation and scatter correction artefacts, and can lead to mis-registration of lesions.^25^ Lesions in the dome of the liver could for example be incorrectly interpreted as being near the base of the lung. The problem can be mitigated using end-expiration breath-hold CT scanning (or quiet tidal respiration CT) combined with respiratory gated PET-CT.^26^ If this approach is taken using a data-driven PET respiratory gating technique, there is no requirement for an external respiratory gating system to be used in any part of the respiratory gated PET-CT acquisition. Data-driven gating for PET can hence be introduced with only minor changes to current workflows.

The current implementation of DDG uses retrospective triggering. At the time of image reconstruction, the DDG-waveform is processed and trigger points are determined. This differs from the prospective gating used by the Real-time Position Management (RPM) Respiratory Gating system (Varian Medical Systems; CA). While prospective triggering is a requirement for radiotherapy treatment purposes, it could lead to inferior performance in the case of PET imaging, especially for irregular respiratory motions that are difficult to predict.^27^ It is hence possible for the RPM device to measure the same waveform as that extracted from the data using DDG, but for the resulting gated images to differ due to differences in the triggering algorithms.

Several studies support the use of respiratory gating for PET-CT.^3, 8,28,29^ Guerra et al^28^ reported the benefits in reporting accuracy and quantification of lung lesions as found from a multicentre, retrospective study comparing 3D PET-CT with 4D PET-CT, for which a cine CT was used to provide phase-matched attenuation correction data. Büther et al^8^ also reported benefits from PET respiratory gating in a prospective study of 74 patients, in which end-expiration breath-hold CT (3D CT) was combined with 4D PET. Improvements in diagnostic image quality, increases in uptake, and decreases in metabolic volumes were reported as compared to 3D PET images. In addition, Büther et al compared the results from respiratory gating using a pressure belt system to those from data-driven gating. Three algorithms were used to generate a DDG signal for each patient (segmented centre of mass, geometric sensitivity, or the Kesner^6 ^algorithm) after which they were scored, and one chosen for use in the 4D PET reconstruction of that patient. Although Büther et al demonstrated similar overall performance between belt-driven gating and data-driven gating, the DDG algorithms used differ substantially from the PCA-based approach used in this work.

## Methods and materials

### Phantom experiments

The Abdo-Man™ phantom,^30^ which is a 3D printed torso phantom with fillable liver and inserts, and on different occasions the NEMA IEC Body Phantom, were filled with F-18, placed on the QUASAR™ respiratory motion platform (Modus QA; London, ON), and imaged using a Discovery D710 PET/CT scanner (GE Healthcare; Milwaukee, WI). Each phantom was filled and scanned on two separate occasions. The total activity in the Abdo-Man phantom at the start of the scan was 5 MBq (0.14 mCi) for scan 1 and 27 MBq (0.73 mCi) for scan 2. For the NEMA IEC Body Phantom, the starting activities were 36 MBq (0.97 mCi) and 43 MBq (1.16 mCi). In all cases, the contrast ratio between hot-spheres and background was 4:1.

The respiratory motion platform was set to be either stationary or to move according to a typical respiratory waveform (the waveform named Typical1, as supplied by the manufacturer). The platform translated the phantom in the Z direction to simulate the cranio-caudal motion of abdominal organs during respiration. The maximum displacement from the central position was set to be ±10 or ±15 mm. Data were hence acquired with maximum displacements between inhalation and exhalation of 0, 20 and 30 mm. The respiratory motion platform includes a stage which rises and falls, in-time with the axial displacement of the phantom. The RPM system (Varian Medical Systems; CA) was used to track the position of a marker that was placed on this stage. A gating signal was also extracted from the raw PET data using the manufacturer’s (GE Healthcare’s) prototype DDG algorithm.

For each phantom experiment, a helical CT scan was performed prior to the collection of 4D PET data at a single bed position. Three repeated acquisitions of 4 min duration were made. Data were then reconstructed using the manufacturer’s Bayesian penalised likelihood algorithm (beta = 400),^31^ with and without quiescent period gating (QPG) for motion compensation.^32^ Approximately 50% of the total emission data were retained within the quiescent period. Data from the phantoms where no motion was applied were also reconstructed using frames of 2 min duration to provide 6 images in total, allowing the effect that acquisition duration has on the percent contrast recovery to be studied.

### Patient scanning

Two retrospective examples of DDG-based QPG applied to patient data are presented, for which informed consent was not required at our institution. The examples, chosen to illustrate the capabilities of DDG, are from the same scanner used for phantom experiments. The indication for PET-CT was evaluation of colorectal cancer metastases within the liver in example 1, and for evaluation of a lung mass in example 2. Each patient fasted for more than 6 h prior to injection of 4 MBq/kg ^18^F-FDG. After a 90-min uptake period, a free-breathing helical CT scan was acquired followed by the collection of free-breathing PET images with 4 min per bed position. Images were then reconstructed in the same manner as that used for phantom studies. Another example has been presented by Morley et al^33^.

### Data analysis

#### Phantom data

The first of the two phantom acquisitions, using ±10 mm displacements, was used to assess the agreement between the gating traces and the driving waveform. The trace created by the PCA-based DDG algorithm was compared to the corresponding trace recorded by the RPM system by first applying a time-shift to the RPM trace for time alignment of the recorded data. This time shift was performed manually via visual inspection, making use of one distinct part of the driving waveform (similar to a cough). The traces were compared for agreement visually and then their correlation was quantified through calculation of Pearson’s correlation coefficient. The traces were then also compared to the driving waveform used to move the motion platform.

After these initial tests of the validity of the DDG trace, the second set of phantom experiments with ±15 mm displacements were used to assess the difference between images reconstructed with RPM- and DDG-based QPG. Images obtained from the phantom experiments were compared visually and quantitatively through analyses of percent contrast recovery (Q) and background variability which followed NEMA definitions.^34^ These analyses made use of circular regions of interest placed in the coronal plane. For the spherical inserts, an ROI with diameter equal to the known inner diameter of the insert was placed at the centre of the sphere as determined visually on each individual PET image. This accounted for the fact that due to respiratory motion, the PET images may not be perfectly aligned with the CT image. 20 circular ROIs of 20 mm diameter were placed in the background region of the phantom, maintaining at least 15 mm distance between the boundary of the ROI and any non-background structure (*i.e.* insert or phantom edge). For the Abdo-Man spherical insert with a solid core, the percent contrast of the cold core was calculated as Q_C_ = (1 – C_C_/C_shell_) × 100%, where C_C_ is the ROI mean value of the cold core, and C_shell_ the ROI mean of the hot shell which excluded the core.

The percent contrast recoveries and background variability were compared for each sphere separately across the four conditions: no motion, RPM-based QPG, DDG-based QPG, and no motion compensation. For each condition and sphere size, the mean contrast recovery was calculated from the three repeated acquisitions. The standard error on the mean was estimated as the standard deviation of the three replicates divided by √3. To test for statistically significant differences, a one-way analysis of variance was performed for each sphere size, followed by Tukey’s Honestly Significant Difference (HSD) post-hoc test to account for multiple comparisons, when indicated at a significance level of 0.05. The software used for statistical analysis was MS Excel 2010 and Python 2.7.

#### Patient examples

Standard uptake values (SUV max) for well-visualised foci in the liver (example 1) or lung (example 2) were reported by a clinician using an Advantage Workstation (v4.6, GE Healthcare; Milwaukee, WI). A coronal slice from each patient is presented.

## Results

**Figure 1.**
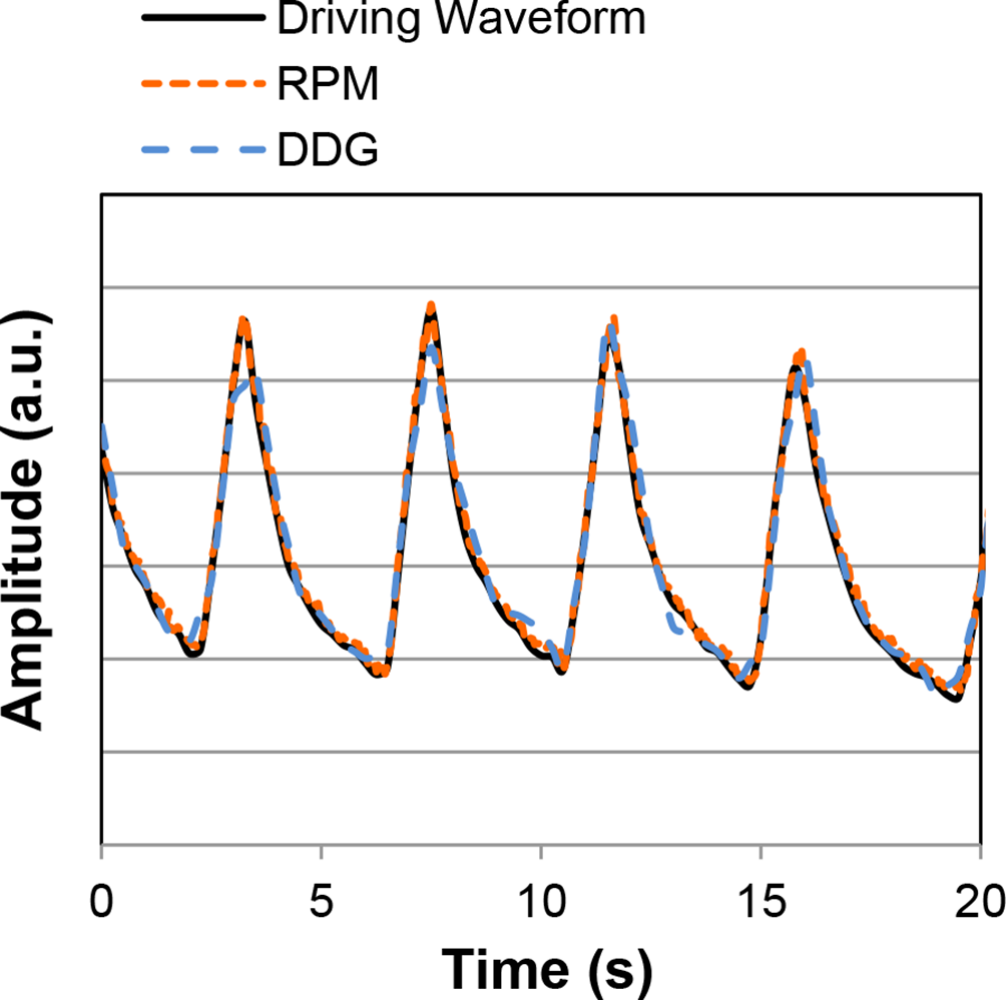
A 20 s portion of the gating trace for Abdo-Man (5 MBq at scan start; ±10 mm motion). The three traces are overlain. The results are representative all phantom acquisitions (both Abdo-Man™ and the NEMA IEC Body Phantom).

For both the Abdo-Man and NEMA IEC Body Phantom, the gating traces produced by the DDG algorithm were in excellent visual agreement with those measured using the RPM system and also with the waveform used to drive the platform. Representative portions of these traces are shown in Figure 1. The calculated correlation coefficients are shown in Table 1.

**Table 1.** Quantitative comparison of gating traces

**Phantom**	**Traces under comparison**	**Pearson’s correlation coefficient**
Abdo-Man; 5 MBq at scan start; ±10 mm motion.	RPM and DDG	0.98
RPM and driving waveform	0.99
DDG and driving waveform	0.97
NEMA IEC Body Phantom; 36 MBq at scan start; ±10 mm motion.	RPM and DDG	0.98
RPM and driving waveform	0.98
DDG and driving waveform	0.99

DDG, data-driven gating; RPM, Real-time Position Management™ system.

**Figure 2.**
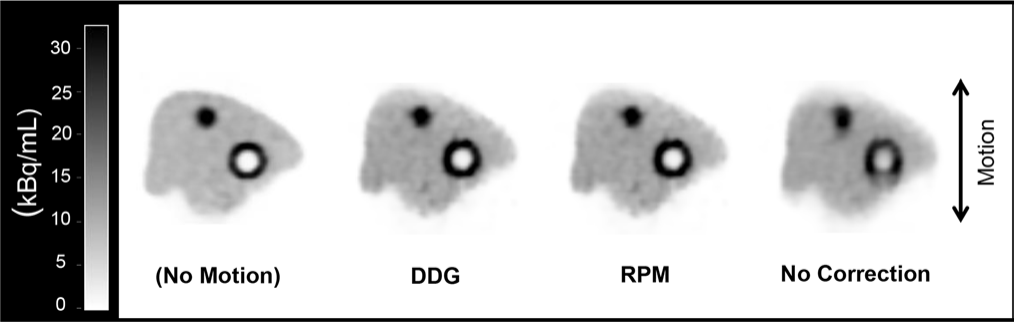
A representative coronal slice from PET images obtained from the Abdo-Man phantom. Images are shown for the case of no applied motion, and for ±15 mm motion with either data-driven quiescent period gating (DDG-QPG), externally driven quiescent period gating (RPM-driven QPG), or no motion correction. DDG, data-driven gating; PET, positron emission tomography; QPG, quiescent period gating; RPM, Real-time Position Management™ system.

**Figure 3.**
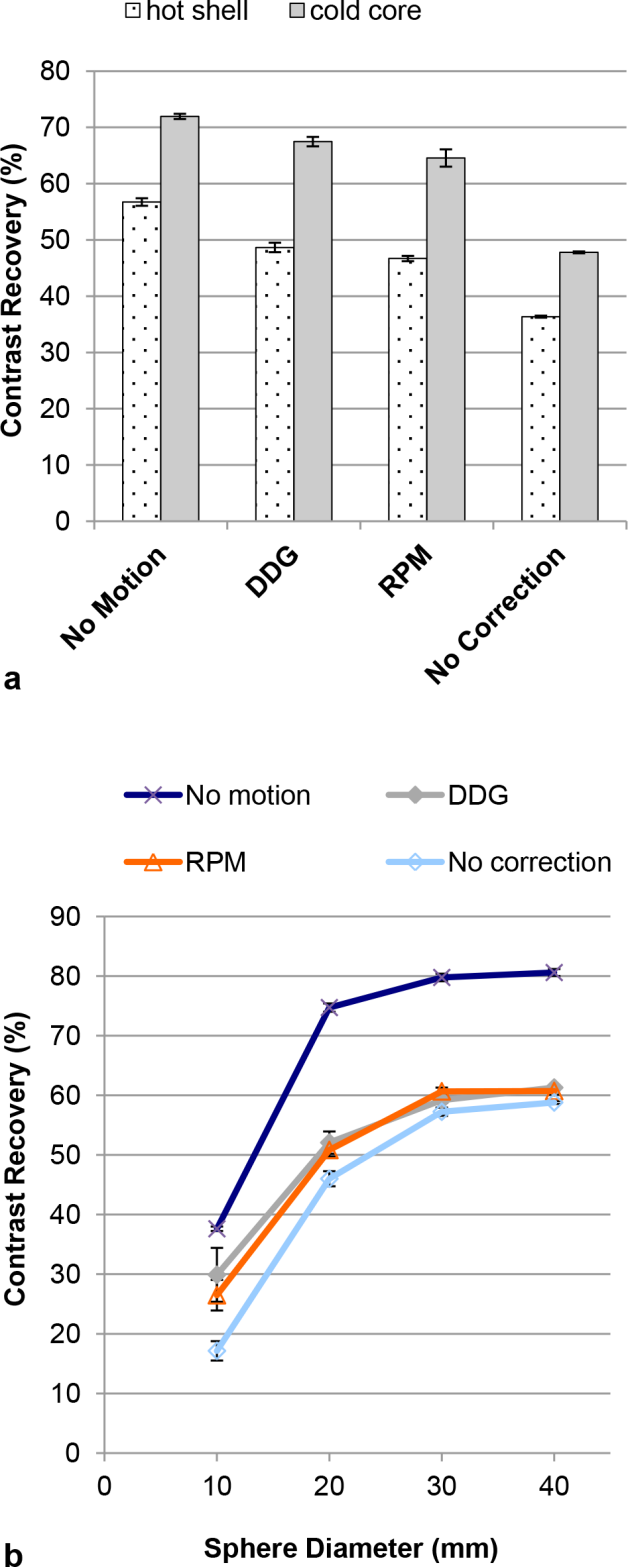
Contrast recovery for different spheres in the Abdo-Man phantom. The plotted data are mean values from three repeated acquisitions. The error bars on each data point represent standard errors on the mean. (a) Results for the sphere with a solid core. (b) Results for the four hot spheres. In almost all cases, contrast recoveries for no motion are significantly greater than those for motion with respiratory gating [data-driven (DDG) or externally driven (RPM)], which are greater than those for motion without gating (all *p* < 0.05). DDG, data-driven gating; RPM, Real-time Position Management system.

**Figure 4.**
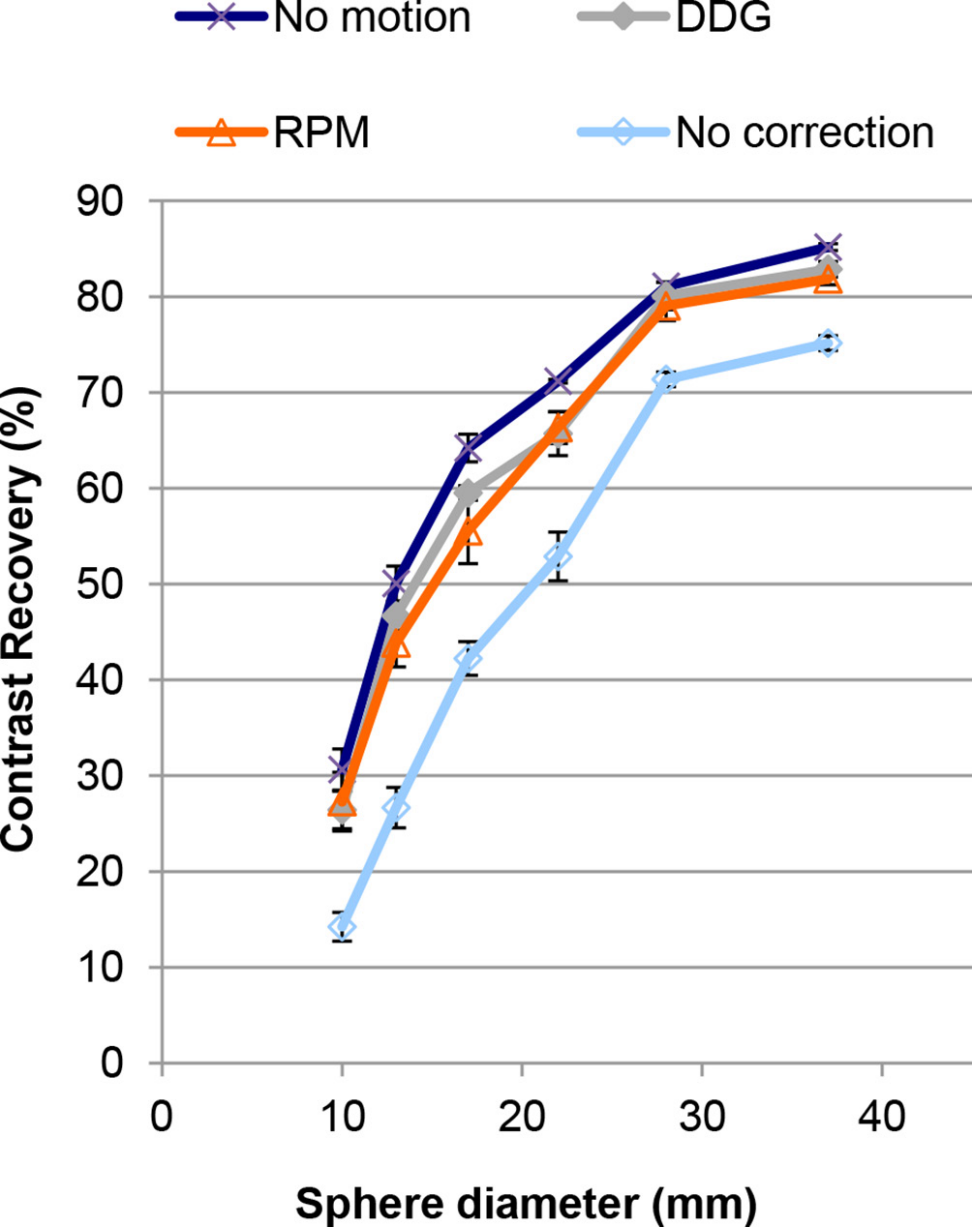
Contrast recovery for the six spheres in the NEMA IEC Body Phantom. The plotted data are mean values from three repeated acquisitions. The error bars on each data point represent standard errors on the mean. In almost all cases, contrast recoveries for no motion are not significantly different from those for motion with respiratory gating (data-driven (DDG) or externally driven (RPM)). Application of motion without gating led to significantly lower contrast recoveries (all *p* < 0.05). DDG, data-driven gating; RPM, Real-time Position Management system.

The simulated respiratory motion led to visible blurring of the PET images in the Z-direction. This was largely recovered through use of DDG- or RPM-based QPG, illustrated by the images in Figure 2. The percent contrasts from these phantom acquisitions are shown in Figures 3 and 4. The quantitative analysis confirms that DDG- and RPM-based QPG provides images with similar levels of motion compensation. In all cases the recovery was increased through the use of QPG, and in all cases the recovery was not significantly different between the DDG- and RPM-based QPG images.

The percent recoveries of the hot shell and the cold core in the Abdo-Man phantom (Figure 3a) were significantly higher when using QPG when compared to no motion compensation, but significantly lower than the case of no motion (all *p* < 0.05; Tukey’s HSD). For the 10, 20, and 40 mm spheres in the Abdo-Man phantom, DDG-based QPG provided significantly higher recovery values compared to no motion compensation (*p* < 0.05; Tukey’s HSD), seen in Figure 3b. For all except the smallest sphere in that phantom, the recoveries from QPG were significantly lower than the recoveries found in the case of no motion (*p* < 0.05; Tukey’s HSD). For the NEMA IEC Body Phantom, QPG provided significantly higher recovery values compared to no motion compensation for all spheres (*p* < 0.05; Tukey’s HSD) as seen in Figure 4. Furthermore, these recoveries were not significantly different from those found in the case of no motion (with the exception of RPM-based QPG for the largest, cold sphere).

The contrast recoveries in both the Abdo-Man phantom and the NEMA IEC Body Phantom were not different when comparing the average of data reconstructed using 2 min frames, with no motion applied, to the same dataset using 4 min frames. The average ratio Q_2min_/Q_4min_ was 1.002 for the Abdo-Man phantom and 1.001 for the NEMA IEC Body Phantom, with no individual sphere size showing a difference of more than 1%.

**Figure 5.**
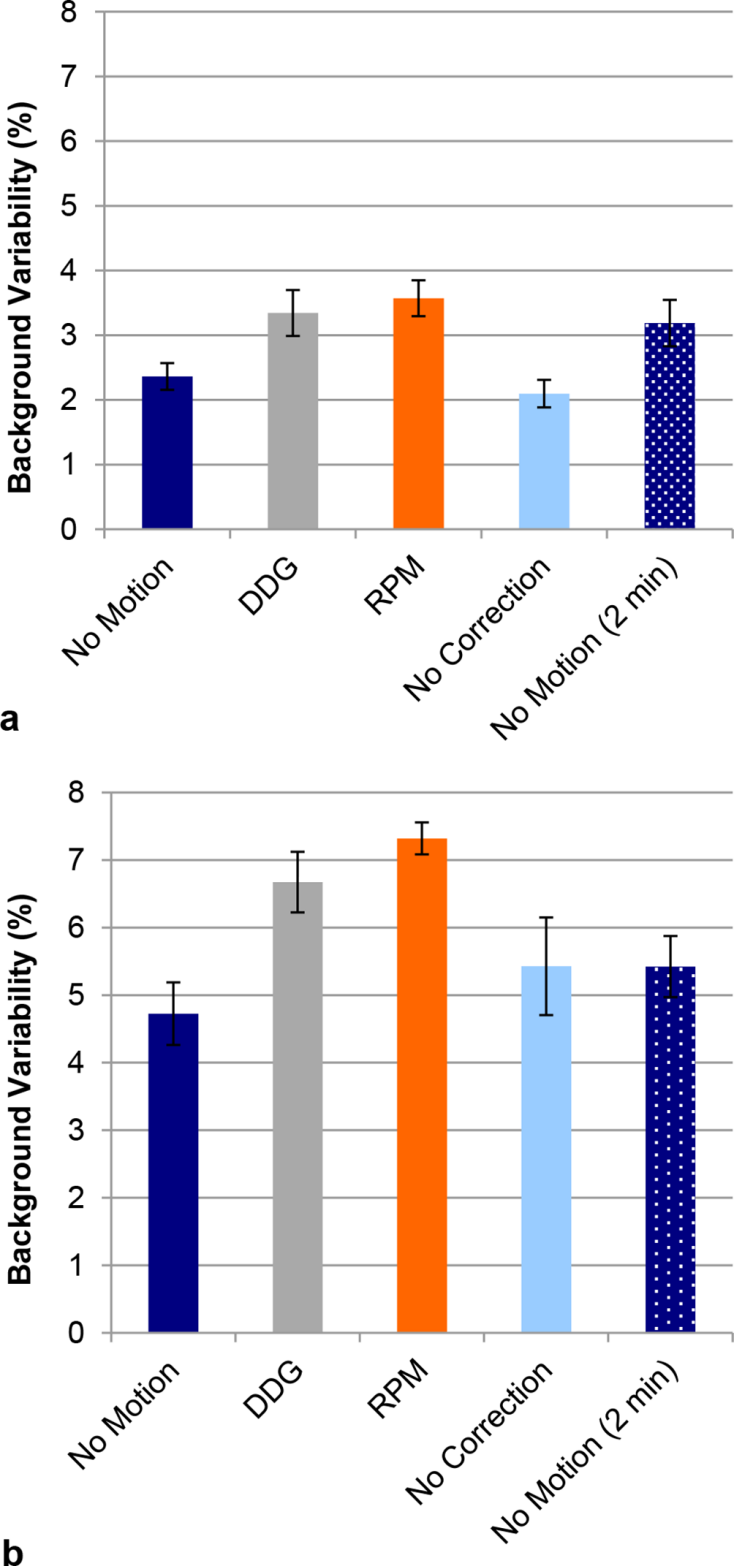
(a) Background variability for 20 mm circular regions in the Abdo-Man phantom. The background variability in the case of data-driven or externally driven gating is significantly higher than the variability without gating (*i.e.* no correction) (*p* < 0.05). (b) Corresponding results for the NEMA IEC Body Phantom. Presented values are the mean from three replicates, with error bars representing the standard error on the mean.

The results for background variability are shown in Figure 5. For the Abdo-Man phantom the variability was significantly higher in the case of RPM- and DDG-based QPG as compared to no motion correction (*p* < 0.05; Tukey’s HSD). There was no significant difference between RPM- and DDG-based QPG, and no difference between no motion compensation and the case of no motion. The results from the NEMA IEC Body Phantom followed a similar trend to those from the Abdo-Man, but the only significant difference was for RPM-based QPG when compared to no motion. The background variability in the NEMA IEC Body Phantom was greater than that for the Abdo-Man phantom, as expected since the activity concentration in the background region of the NEMA IEC Body Phantom was approximately half that of the Abdo-Man phantom.

**Figure 6.**
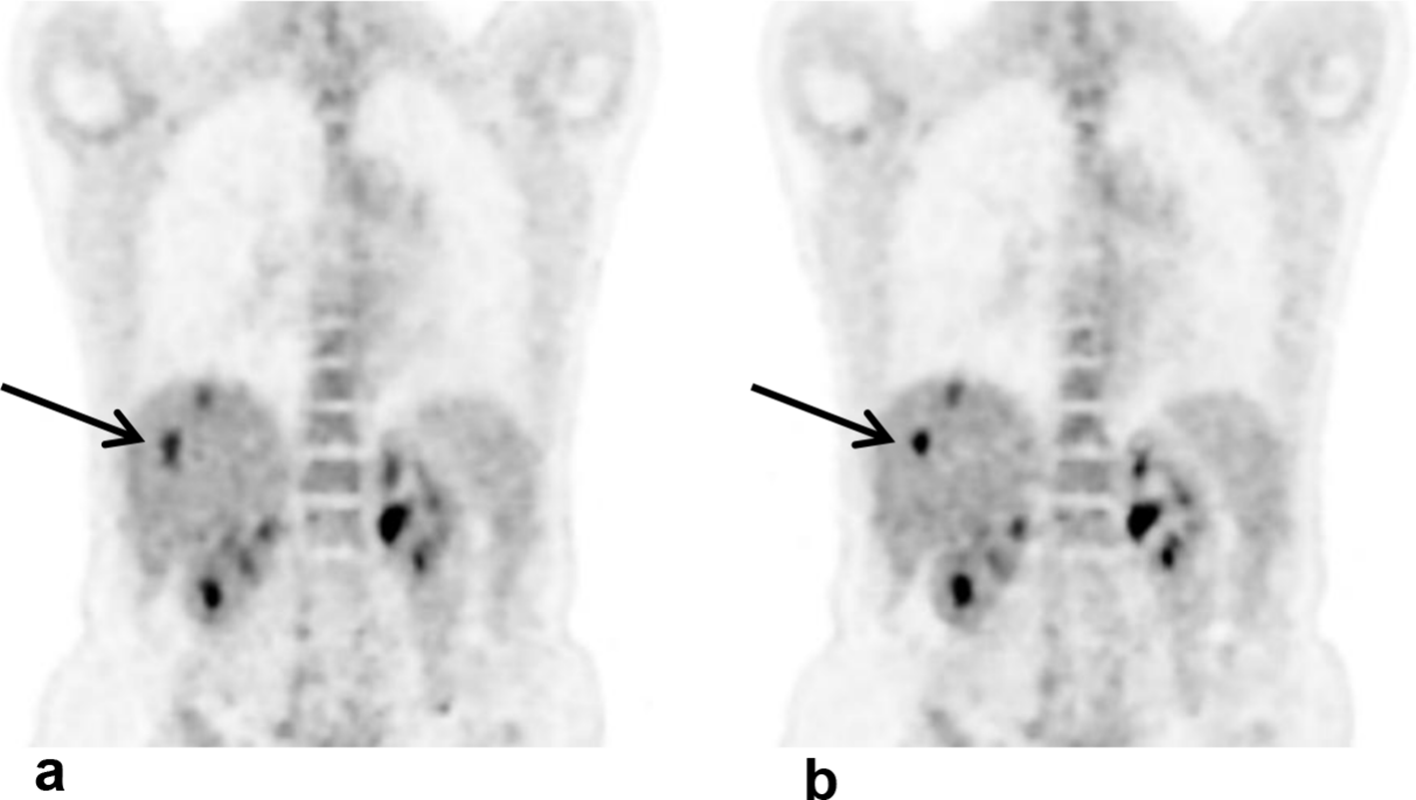
Example images from a patient demonstrating the improved contrast and visual image quality that can be obtained using respiratory gating. A coronal slice is shown. (a) Without motion compensation (using 2 min of data). (b) With data-driven quiescent period gating (using 4 min of data, reduced to approximately 2 min after gating). The linear grey scale is from an SUV of 0–6. SUV, Standard uptake value.

**Figure 7.**
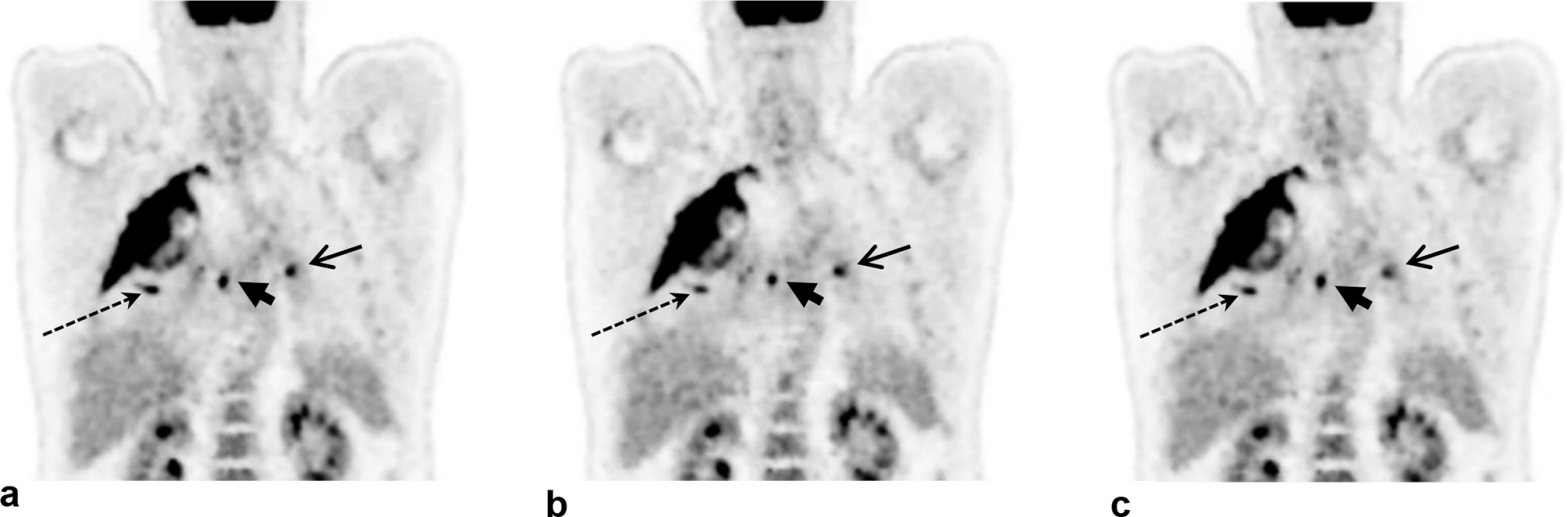
Example images from a patient demonstrating the improved contrast and visual image quality that can be obtained using respiratory gating. A coronal slice is shown. (a) Without motion compensation (using 2 min of data). (b) With data-driven quiescent period gating (using 4 min of data, reduced to approximately 2 min after gating). (c) With externally driven (RPM) Quiescent Period Gating (using 4 min of data, reduced to approximately 2 min after gating). The linear grey scale is from an SUV of 0–6. SUV, Standard uptake value.

The first patient example showing DDG-based QPG is provided in Figure 6. The example demonstrates improved clarity of lesions with the application of DDG-based QPG, as compared to no motion compensation with a matched count-level. The lesions appear more compact with QPG, in agreement with an observed increase in the SUV of a prominent liver lesion; the lesion had an SUV max of 6.4 without motion compensation, increasing to 10.1 after DDG-based QPG. A second example is provided in Figure 7, for which both DDG and RPM-based gating were available and are presented alongside the non-gated image. This example demonstrates improved clarity of thoracic lesions with reduced blurring in the cranio-caudal direction. The SUV max from three distinct areas of focal uptake, shown in Figure 7 by a long dashed arrow (pulmonary nodule), a short arrowhead (right hilar nodes), and an arrow (left hilar nodes), was measured in the non-gated image, the DDG-based QPG image and the RPM-based QPG image. For the pulmonary nodule, SUV max values were 9.4, 11.1 and 10.8 respectively. For the right hilar nodes the values were 9.9, 9.5 and 10.0. For the left hilar nodes the values were 7.6, 7.8 and 5.8.

## Discussion

Our results demonstrate strong association and excellent visual agreement between the gating signals generated by DDG and those measured using an external, camera-based device. This agreement was found using the NEMA IEC Body Phantom and for the Abdo-Man anthropomorphic phantom. Analysis of images created using QPG verified that these gating signals are appropriately triggered to provide images in which the effects of respiratory motion are mitigated. Visual analyses were confirmed by quantitative measures of recovery, which found DDG- and RPM-based QPG to perform similarly, and for both to provide superior recovery values as compared to no motion compensation. There was however some motion within the 50% of retained data used for QPG, and this is likely the reason for which the QPG images were inferior to those obtained when no motion was applied to the phantom. This is not a feature of DDG *per se*, but a feature of the chosen method of respiratory compensation. The reduced number of coincidences retained in the QPG dataset could also negatively affect the measured contrast recoveries, but the results found for the 2 min duration frames, having similar contrast to 4 min frames, implies that this was not the case for these datasets.

The example provided in Figure 6 demonstrates that respiratory gating may have a large impact for liver lesions, owing to the fact that respiratory motion extends beyond the lungs. We plan to assess the benefit of data-driven respiratory gating for lesions in the liver and other abdominal organs in a future study. A previous study making use of an external gating system found respiratory gating to be beneficial to image quality for liver lesions, with increased SUV max and decreased metabolic tumour volumes.^29^

Occasionally, a small discrepancy between the DDG trace and the driving waveform was noticed, as seen at the second peak in Figure 1. We consider the small discrepancies at some peaks and valleys to be sufficiently small to not impact the performance of respiratory gating. It is possible that these discrepancies, which correspond to 1–3 mm, were caused by a limitation of the mechanics of the respiratory motion platform if the phantom stage was not moved exactly as intended when the direction of motion was switched.

Although there are many evaluations of respiratory gating techniques for PET in the literature, most of these do not report phantom data in which the ground truth was known. Simulations validated by phantom experiments performed by Liu et al^35^ found that respiratory motion can lead to reductions of around 30% in SUV max for liver and lung lesions, with increases in lesion volumes. Bowen et al^36^ found respiratory motion applied to a phantom to cause a decrease of 20% in the ratio of hot sphere SUV max to background. In the current study we quantified contrast recovery based on SUV mean rather than SUV max, and found reductions of nearly 50% when comparing motion (without correction) to no motion. Ren et al^11^ evaluated a data-driven gating technique on 10 patients and compared the data-driven respiratory waveform to that provided by a pressure belt system. The correlation coefficient between the two varied between patients, averaging 0.79 with a maximum of 0.89. Kesner et al^6^ evaluated a data-driven method on 22 subjects and found average correlation coefficients of <0.6. These can be compared to the correlation coefficients of >0.97 found in the current work for the case of a moving but rigid phantom.

The use of phase-matched gating to create a sequence of images across the respiratory cycle, followed by non-rigid registration and addition, could mitigate the respiratory motion to a greater extent and will be investigated in future studies. Such an approach was not chosen for the current evaluation, as a robust comparison between DDG and RPM was the main focus of this investigation, as opposed to assessing the optimal method of respiratory motion correction. Furthermore, quiescent period gating is available on many PET systems.

The results for background variability were as expected based on the effective acquisition time, and thus number of coincidences, for each of the four conditions. Both QPG images were reconstructed from only half of the acquired coincidences, and hence the background variability increased. This increase was to a level consistent with the background variability found when the no-motion data were reconstructed using 2-min frames.

One of the benefits of using DDG as opposed to an external gating system is in the time that can be saved; there is less time required to train staff in the use and setup of equipment, there is no need to coach the patient or explain the workings of an external system, and there is no time spent in setting up a gating device for each patient. Patient comfort may also be improved. Another benefit of DDG is the inherent time-synchronisation with the scanner, which can be a source of significant error if external gating systems are mis-calibrated.^8^ External gating systems are also subject to operator-based errors, which can occur in a significant proportion of patients (*e.g.* 13%),^37^ typically yielding no suitable respiratory signal.

A limitation of PET DDG is that this approach does not provide a gating signal for 4DCT. While 3DCT using either shallow breathing or a breath-hold technique is acceptable for most clinical applications, and impart less radiation dose than 4DCT, they do not provide phase-matched attenuation correction data. Note however that it may be possible to re-align a breath-hold CT to each phase of the 4D PET when this is desired.^37^ As PET DDG methods such as the one investigated here operate on the PET data, they are transferable to PET-MR systems.^21^

The current evaluation of DDG has some limitations, notably it is performed primarily using phantoms. These phantoms are useful due to the fact that the driving waveform was both realistic and known, the phantoms themselves were reasonably anthropomorphic, and the ground truth for the radioactivity distribution was also known. However, the phantom motion still represents rigid-body motion which is not typically the case in human respiration. For patients one can compare DDG to RPM or another external gating system, but these external systems are not always reliable. Evaluations of image quality and the quantification of lesions within large patient cohorts, combined with the success rates for DDG in such groups, will be performed in future work.

## Conclusion

A data-driven gating method for PET, based on principal component analysis of the raw PET data, was evaluated and found to provide a reliable respiratory gating signal in anthropomorphic phantom studies and in example patients. The method obviates the need for external gating equipment, and may enable respiratory gating to become routine practice for clinical PET-CT in the near future.
